# Amplitude-modulated cold atmospheric pressure plasma jet for treatment of oral candidiasis: *In vivo* study

**DOI:** 10.1371/journal.pone.0199832

**Published:** 2018-06-27

**Authors:** Aline Chiodi Borges, Gabriela de Morais Gouvêa Lima, Thalita Mayumi Castaldelli Nishime, Aline Vidal Lacerda Gontijo, Konstantin Georgiev Kostov, Cristiane Yumi Koga-Ito

**Affiliations:** 1 Department of Environmental Engineering and Oral Biopathology Graduate Program, Institute of Science and Technology, São Paulo State University (UNESP), São José dos Campos, Brazil; 2 Department of Chemistry and Physics, Guaratinguetá Faculty of Engineering, São Paulo State University (UNESP), Guaratinguetá, Brazil; University of Pittsburgh, UNITED STATES

## Abstract

The aim of this study was to establish an effective and safe protocol for *in vivo* oral candidiasis treatment with atmospheric plasma jets. A novel amplitude-modulated cold atmospheric pressure plasma jet (AM-CAPPJ) device, operating with Helium, was tested. *In vitro* assays with *Candida albicans* biofilms and Vero cells were performed in order to determine the effective parameters with low cytotoxicity. After the determination of such parameters, the protocol was evaluated in experimentally induced oral candidiasis in mice. AM-CAPPJ could significantly reduce the viability of *C*. *albicans* biofilms after 5 minutes of plasma exposure when compared to the non-exposed group (p = 0.0033). After this period of exposure, high viability of Vero cells was maintained (86.33 ± 10.45%). Also, no late effects on these cells were observed after 24 and 48 hours (83.24±15.23% and 88.96±18.65%, respectively). Histological analyses revealed significantly lower occurrence of inflammatory alterations in the AM-CAPPJ group when compared to non-treated and nystatin-treated groups (p < 0.0001). Although no significant differences among the values of CFU/tongue were observed among the non-treated group and the groups treated with AM-CAPPJ or nystatin (p = 0.3201), histological analyses revealed marked reduction in candidal tissue invasion. In conclusion, these results point out to a clinical applicability of this protocol, due to the simultaneous anti-inflammatory and inhibitory effects of AM-CAPPJ with low cytotoxicity.

## Introduction

*C*. *albicans* is considered the most frequent fungal species in human microbiota and colonizes approximately 50% of healthy individuals [1]. Due to its opportunistic nature, under favorable conditions, *C*. *albicans* can express virulence features and cause infections. These conditions are host-related and are usually associated to imbalance in the anatomical barrier function or in the commensal microbiota [2]. The occurrence of infection depends on a complex interplay between fungal virulence factors and the immune system of the host [3].

Oral candidiasis is a common occurrence among HIV-positive patients [4], and it is reported as one of the first clinical manifestations of HIV infection [5]. Organ transplanted patients, elderly, diabetics and patients under corticosteroids or long-term antimicrobial therapy are also prone to develop *Candida* infections [6–10]. In severe cases, superficial infections can be the first step to homogeneously disseminated fungaemia [11].

The treatment of fungal infections represents a challenge in medical areas. Increasing in antifungal resistance rates has been reported over the last decades [12]. It is known that some *Candida* species show intrinsic resistance and others have high ability to develop resistance [13]. Multidrug resistance in different *Candida* species has also been reported and it is considered an emergent problem [14]. It is noteworthy that the adverse effects of the antifungal drugs, such as toxicity and interactions with other drugs, are also relevant problems in this context [15].

In addition, the number of new therapeutic options for candidiasis remains limited. The discovery of molecular targets that can be used for drug development is challenging due to the similarity between fungi and human cells [16]. Combinations of conventional antifungal drugs and plant-derived compounds [4] as well as the associations among drugs [14] have been proposed as alternatives to overcome this challenge. Photodynamic inactivation has been also reported as a viable alternative for oral candidiasis [17].

Cold atmospheric pressure plasmas have emerged as a promising and innovative technology for biomedical applications in the last years [18]. Although the exact mechanism of microbial inactivation has not been fully understood yet, it has been suggested that the effect of plasma is related to the presence of reactive oxygen and nitrogen species (RONS) that act in a synergic way resulting in antimicrobial activity [19]. Cold atmospheric pressure plasma showed inhibitory effect on *Candida* planktonic cultures and biofilms and also enhanced the effect of conventional antifungal drugs [20–23].

The proposal of a new antimicrobial therapy should be based on its efficiency, associated to minimal effects on the host. Despite the reports on antifungal effects, the cytotoxicity of cold plasma jets on mammalian cells has scarcely been explored in the literature. Low toxicity was reported after 30 seconds of plasma jet exposure [24]. However, this period of exposure is much lower than the period of 300 seconds, usually needed to affect the viability of *C*. *albicans* biofilms [22, 25].

A previous study showed that a cold atmospheric pressure plasma jet operating with Helium at a continuous sinewave voltage signal could disrupt *C*. *albicans* biofilm from 150 seconds of exposure [26]. However, at this period of exposure, the plasma jet was cytotoxic to Vero cells *in vitro* [26]. These results pointed out to the need of a safe protocol for the treatment of candidiasis *in vivo*. To reach the purpose of the study, an extensive *in vitro* investigation was done to determine effective parameters against *C*. *albicans* biofilm with low cytotoxicity to Vero cells, by using an amplitude modulated cold atmospheric pressure plasma jet device operating with Helium (AM-CAPPJ). The protocol was subsequently evaluated in experimentally induced oral candidiasis in mice.

## Material and methods

### Amplitude modulated cold atmospheric plasma jet (AM-CAPPJ) device

**Fig 1:**
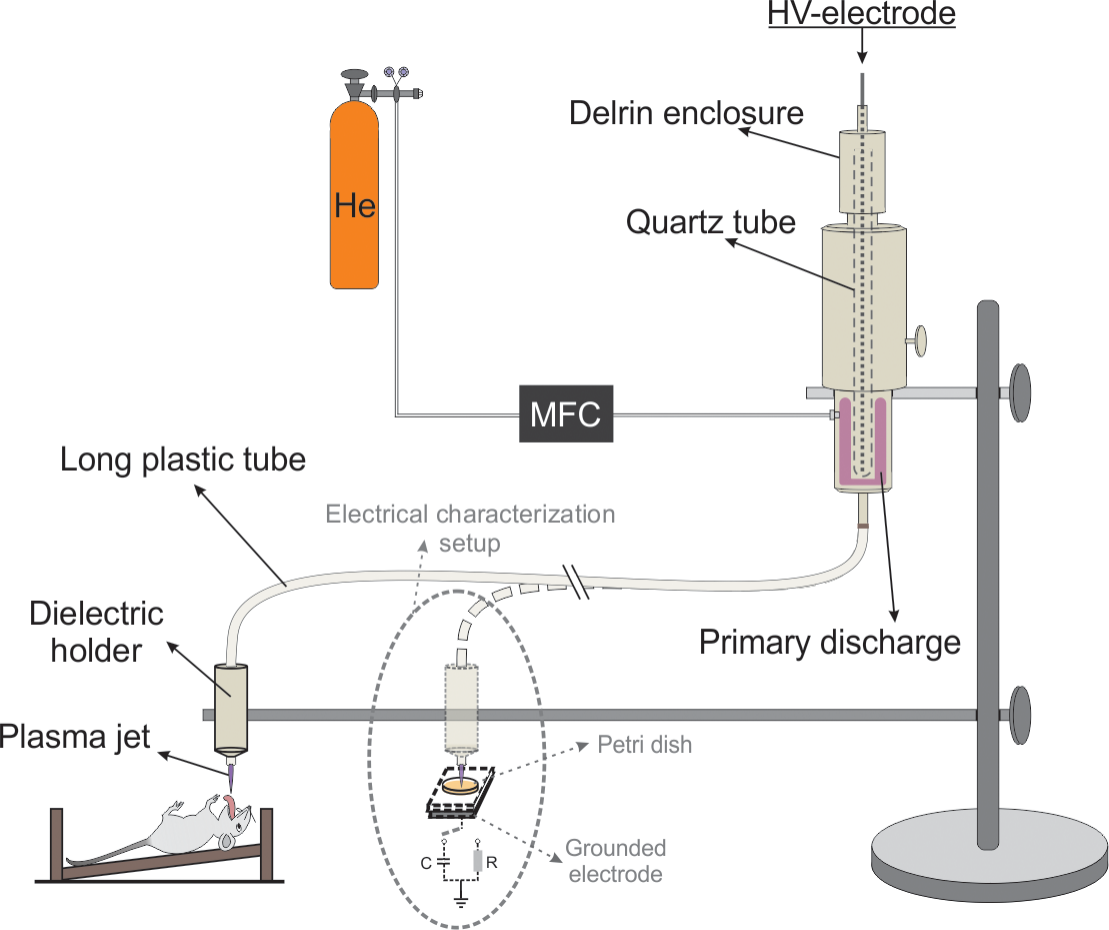
Experimental setup.

The plasma jet device employed in this study consists of a syringe-like dielectric enclosure, with a high-voltage pin electrode, embedded into a close-ended quartz tube, placed in its central axis. Fig 1 shows a schematic layout of the experimental setup used for *in-vivo* treatments. Also, an inset in the design shows the target adaptation that was used for plasma jet electrical characterization.

The gas, 99.5% purity Helium (Air Liquide, Brazil) from a gas cylinder was fed into the system at a constant flow rate of 2.0 SLM using a mass flow controller (N100 Horiba STEC, Japan). Differently from most plasma jet devices, where the plasma from the dielectric enclosure is injected directly into ambient air, here the reactor nozzle was connected to a 1.0-m-long, 2.5-mm-diam polyurethane tube (Kangaroo^TM^ Nasogastric Feeding Tube, 12 Fr/Ch).

Inside the tube was installed a thin metal wire (Φ = 0.1 mm) at floating potential. The wire penetrates few mm inside the dielectric enclosure and terminates 2 mm before end of the plastic tube. When high voltage (HV) is applied to the encapsulated pin electrode inside the syringe, dielectric barrier discharge (DBD) is ignited inside the syringe inducing charges on the floating wire in the plastic tube. As shown in a previous study [27] the resulting electric field at the downstream wire tip is high enough to generate plasma, which is launched outside the plastic tube end forming a small “secondary” plasma jet. More information about this arrangement and its operation principle can be found in previous works [27, 28]. As already shown [27] for biomedical applications tube length of 1.0 m is the best choice due to the following reasons. Firstly, the secondary plasma jet is ignited and it is produced far from the high-voltage generator thus reducing risk of electric shock to the target. Secondly, the potential on 1-m-long wire embed in polyurethane tube is sufficiently low so that one can hold the tube and manipulated it by bare hand. Finally, the power of the plasma jet produced at end of the plastic tube is on the order of couple of Watts [28], which makes it suitable for biomedical applications. Yet, for treatment of living tissues a precise control of the plasma jet parameters is necessary, which was achieved in this study by applying an amplitude modulated voltage signal.

The plasma jet device was powered by a Minipuls4 AC power supply (GBS Elektronik GmbH, Germany) capable of generating an AC voltage signal with amplitude up to 20.0 kV and frequency between 20 and 40 kHz. To adjust accurately the plasma plume power, the applied voltage signal used in this study was amplitude modulated. In this mode of operation, a burst of 10 cycles of HV signal with frequency of 32.0 kHz and amplitude of 13.0 kV is followed by voltage off period. This sequence is repeated with a repetition period of 1.5 ms resulting in a voltage duty cycle of 22%. Voltage amplitude was measured at HV generator by voltage divider 1:2000 and a detailed view of the amplitude modulated voltage signal was published elsewhere [28]. To record discharge current waveform, the plasma plume was directed to a grounded electrode covered by 4-mm-thick glass slab (see the inset in the Fig 1). The discharge current was calculated from the voltage drop across a serial resistor of 105 Ω. To measure transferred charge in the discharge, the resistor was replaced with a capacitor of 10 nF. The power was calculated using the Lissajous figure method. The distance between the plastic tube tip and the surface to be treated was set at 1.5 cm. During the plasma treatment, sample temperature was monitored by an infrared thermometer (Daie, model GM-300).

The AM-CAPPJ configuration and the results of optical emission spectroscopy were presented in a previous work [28]. The spectra of the plasma plume generated at the end of the plastic tube exhibited atomic oxygen emission lines and characteristic molecular bands of N_2_ and N_2_^+^ (second positive and first negative systems, respectively), evidencing the presence of reactive oxygen and nitrogen species (RONS) [28]. These species can diffuse radially outward and cover distances larger than the plasma jet size [23]. Since *C*. *albicans* biofilms in this study are quite small (limited by the size of well in microtiter plate and the mouse tongue) there was no need for the plasma plume manipulation during the treatment and the jet was kept static at normal incidence on the surface.

### Effect of AM-CAPPJ on *C*. *albicans* biofilms *in vitro*

*C*. *albicans* ATCC 18804 was stored in Brain and Heart Infusion (BHI) broth with 20% of glycerol at -80°C until use. After defrosting, *C*. *albicans* cells were grown in Sabouraud dextrose agar at 37°C for 24 h. Standardized fungal suspensions containing 10^6^ cells ml^-1^ in physiologic solution (NaCl 0.9%) were obtained by using a spectrophotometer.

Biofilms of *C*. *albicans* ATCC 18804 were formed at the bottom of 96-well microplates. RPMI 1640 (with L-glutamine, without bicarbonate, buffered to pH 7.0 with MOPS) medium supplemented with 2% glucose (200 μl) containing 10^4^ cells of *C*. *albicans* was added to each well. After 90 min of incubation (37°C; 80 rpm), the culture medium was removed and the wells were washed twice with physiologic solution (NaCl 0.9%) to recover non-adherent cells. Then, 200 μl of fresh RPMI medium were added to each well and the plates were incubated for 24 h. The culture medium was subsequently removed; the biofilms were washed twice and exposed to AM-CAPPJ for 2.5, 5 and 7.5 min. The cell viability was determined after serial dilution and inoculation on Sabouraud dextrose agar. The results were expressed as colony-forming units per biofilm (CFU/biofilm) values after incubation at 37°C for 48 h. Three independent experiments were performed in triplicate.

### Cytotoxicity of AM-CAPPJ *in vitro*

The cytotoxic effect of plasma jet was determined as described previously [26]. Vero cells (ECACC 84113001) were cultivated in Dulbecco's Modified Eagle's medium (DMEM) supplemented with 10% of fetal bovine serum, 100 IU mL^-1^ of penicillin, and 100 μg ml^-1^ of streptomycin and grown at 37°C in an atmosphere of 95% air and 5% CO_2_. The cells (6 x 10^4^/well) were cultured for 24 h, the culture medium was then removed and 30 μl of Hanks' Balanced Salt Solution at pH 7.4 with 10 mM 4-(2-hydroxyethyl)-1-piperazineethanesulfonic acid were added. The cells were exposed to AM-CAPPJ jet for 3 and 5 min.

The cell viability was determined immediately, 24 and 48 h after plasma jet exposure using MTT assay. For the analyses of later effects (24 and 48 h), the culture medium was immediately added after plasma jet exposure. The percentage of cell viability was obtained considering the non-treated group as 100%. A reduction of cell viability between 70% and 50% comparing to non-treated group was considered of moderate cytotoxicity [29]. The experiments were performed in triplicate in two independent experiments.

### Treatment of experimentally induced oral candidiasis with AM-CAPPJ

This study was carried out in strict accordance with applicable national and international guidelines. The protocol was approved by the Committee on the Ethics of Animal Experiments of the Institute of Science and Technology, São Paulo State University (protocol Number 01/2015). Surgical procedures were performed under anesthesia, and all efforts were made to minimize suffering.

**Fig 2:**
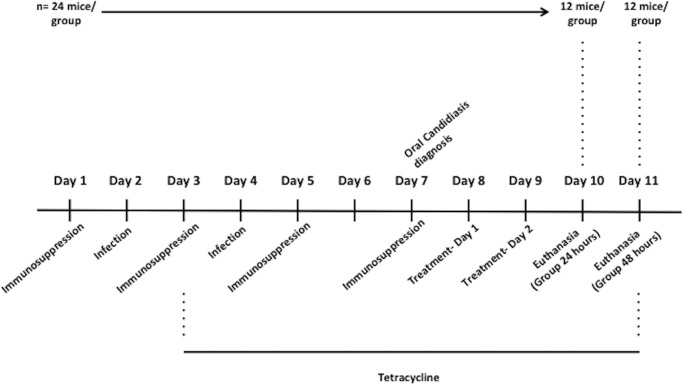
Timeline of the experiment.

During the experiment, mice were fed with regular diet and water supplemented with tetracycline (Terracimina–Pfizer; 0.83 mg/ml) *ad libitum*. They were kept in ventilated cages in a facility with regular (12-hour) dark-light cycle. Seventy-six mice (*Mus musculus*, Swiss), weighing approximately 30 g, were included in the study. Oral candidiasis was experimentally induced as described by Rossoni et al. [30] and the experimental phases are illustrated in Fig 2.

**Fig 3:**
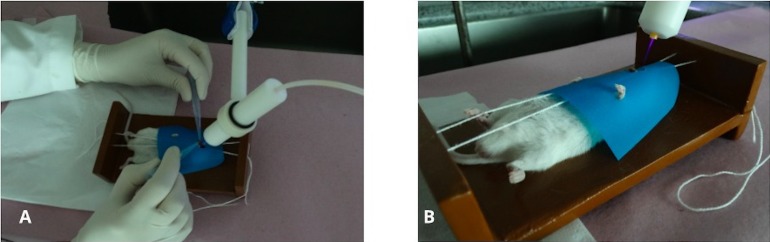
Photos of (A) experimental arrangement for in vivo tests. (B) In vivo application of plasma jet.

Methylprednisolone suspension (100 mg/Kg; Depro-Medrol, Pfizer, Belgium) was administrated intraperitoneally for 4 days. *C*. *albicans* infection was induced twice within a 48-hour interval. For this purpose, all animals were anesthetized using injection of ketamine base (100–150 mg/kg; Ceva, Brazil) and xylazine hydrochloride (10–15 mg/kg; Ceva, Brazil). Then, swabs immersed in *C*. *albicans* ATCC18804 suspension (1 x 10^8^ cells ml^-1^) were maintained in contact with the mice’s tongue for 3 minutes. Seventy-two hours after the last inoculation, oral candidiasis was clinically diagnosed. Mice that developed lesions were randomly divided into three groups (n = 12 each group): Control group (non-treated), group treated with plasma jet, and group treated with nystatin (100,000 UI, Neo Química, Brazil). The experimental arrangement of AM-CAPPJ for in vivo tests is illustrated in Fig 3.

The treatments started 24 hours after the diagnosis of oral candidiasis. The lesions were treated with AM-CAPPJ for 5 min in two consecutive days. Nystatin was applied directly on the lesion once a day for 2 days. Twelve animals per group were sacrificed after 24 or 48 hours after the end of the treatment. Euthanasia was performed by injection of overdosed anesthetic solution. Five tongues per group were histologically analyzed and seven were used for fungal CFU counts.

To evaluate the possible effect of AM-CAPPJ on the tongue’s tissues, a group of mice without *C*. *albicans* infection was submitted to plasma jet treatment (n = 4).

Histological analyses were performed to evaluate the effect of each treatment. Five animals of each subgroup (24 or 48 hours) had their tongues removed, immersed in 10% formalin fixative, washed, processed and paraffin embedded. Semi-serial 5 μm sections were obtained and stained with PAS (Periodic acid- Schiff) and HE (Haematoxylin-Eosin). Two random sections, in different depths, were analyzed.

*C*. *albicans* epithelial invasion was analysed in PAS stained slides. Twenty-one histologic fields were analysed per section, leading to 42 fields/tongue. The semi-quantitative analysis was performed at 400x magnification using an optical microscopy (Zeiss) and the tissue invasion was determined by counting the invading hyphae. The counts were scored as follows: 0: hyphae absence; 1: 1–5 hyphae; 2: 6–15 hyphae; 3: 16–50 hyphae; and 4: more than 50 hyphae [31].

Semi-quantitative and descriptive analyses of the tissue inflammatory response were performed in HE slides. Scores were attributed according to the number and type of inflammatory epithelial alterations (6 fields/tongue). The fields were analyzed at 200x and 400x magnification. Presence of hyperkeratosis, hyperplasia, acanthosis, exocytosis, spongiosis, alteration in the basal layer pattern, loss of lingual papillae, and presence of intraepithelial microabscess was assessed. The given scores are described on Table 1.

**Table 1:** Scores for epithelial alterations.

Score	Characteristic
1	Hyperkeratosis and/or hyperplasia
2	Hyperkeratosis + other 2 inflammatory alteration
3	Hyperkeratosis + other 3 inflammatory alterations
4	Intraepithelial microabscess + any other inflammatory alteration

For microbiologic analyses, 7 animals of each group were randomly selected. Tongues were removed and macerated in sterile physiologic solution (NaCl 0.9%). The suspensions were plated in Sabouraud dextrose agar supplemented with chloramphenicol.

## Results

### Plasma treatment

**Fig 4:**
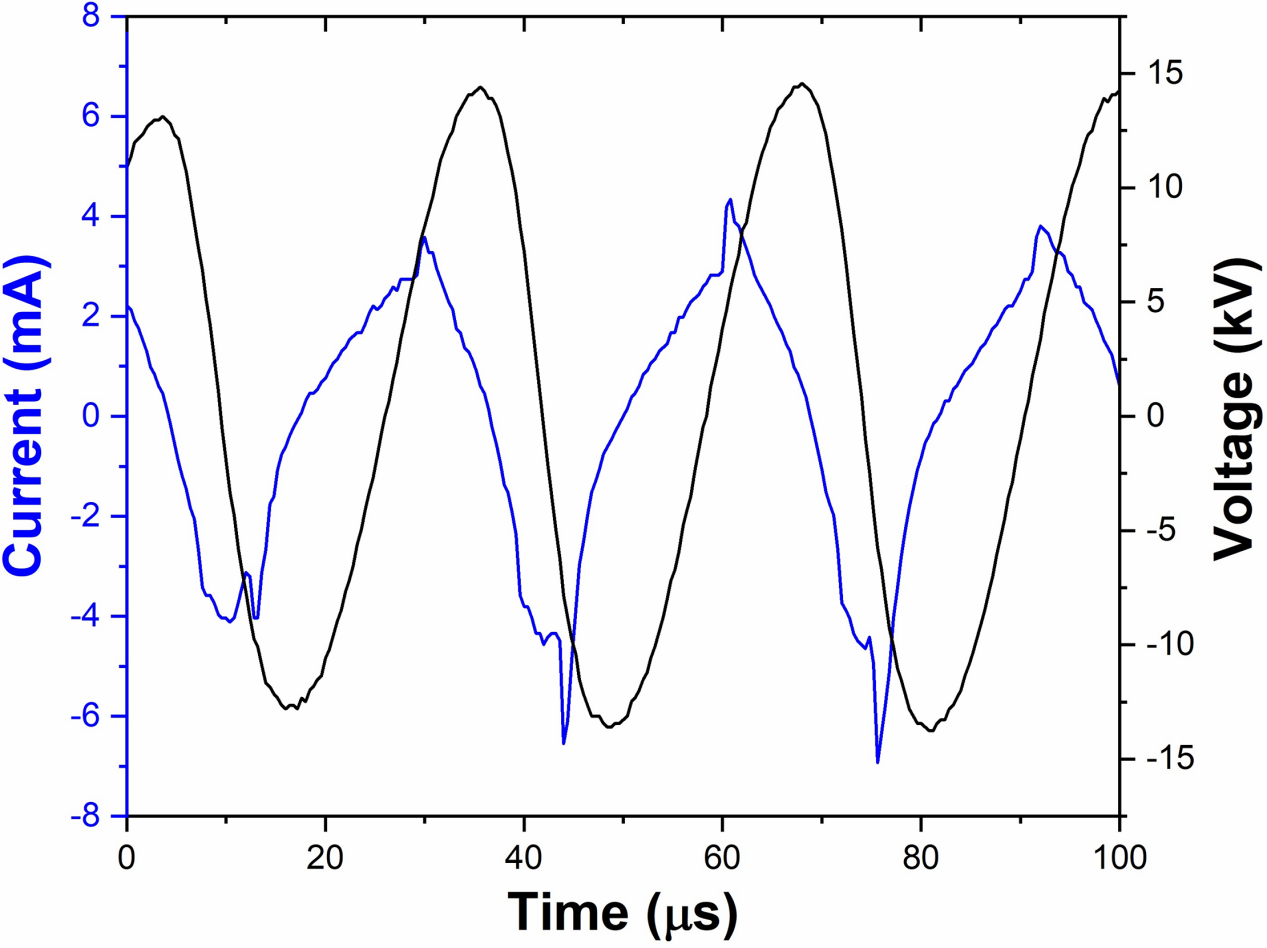
Typical current and voltage waveforms for 2.0 slm of He flow and 1.5 cm distance to the target.

Plasma treatments (*in-vitro* and *in-vivo*) were conducted using AM-CAPPJ produced at the end of 1-m-long plastic tube, whose schematic layout is shown in Fig 1. Fig 4 depicts typical current and voltage waveforms in a timeframe of 100 μs taken within a HV oscillation burst.

**Fig 5:**
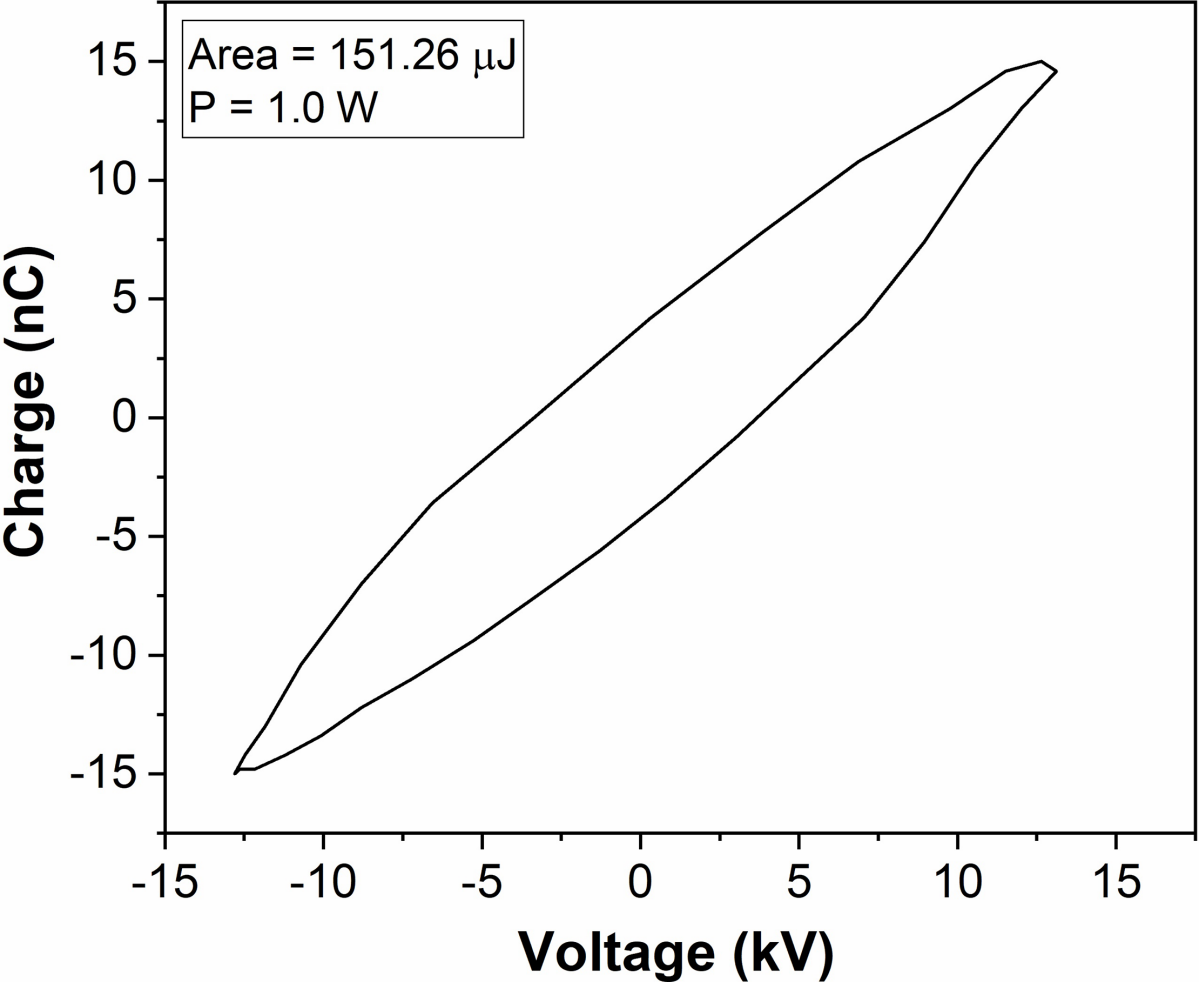
Q-V Lissajous figure for one voltage cycle. The figure’s area is 151.3 μJ.

As can be seen, the voltage signal within a burst is a slightly irregular sinewave with period of 31.0 μs and amplitude of 13.0 kV. The discharge current waveform is similar to the one observed in other works [28, 32]. It consists of single current peaks appearing in each half-cycle of the voltage that are superimposed to a capacitive current. A typical Q-V Lissajous figure for a voltage period is presented in Fig 5.The area of this figure gives the discharge energy ***E***_***c***_ per a cycle of voltage oscillation. Then the mean power consumed in the discharge ***P*** can be calculated as:

$$P=E_{c}fD$$

Where ***f*** and ***D*** are the frequency and the duty cycle of the voltage signal, respectively. More information about the plasma jet power in burst mode of operation and how it scales with the duty cycle of the applied voltage can be found in a previous work [28].

For the experimental conditions used in this work, the total discharge power (the power of the primary DBD discharge plus the power of the plasma plume) was 1.0 W. From this power, about 40% are due to the primary DBD while 60% are consumed in the plasma plume generated at the tip of the plastic tube [28]. Therefore, the mean power delivered to the sample was around 0.6 W. An important parameter to evaluate treatment efficiency and compare it to other works is the energy density [32] or, as for our case, energy per unit area. For *in-vivo* treatment the area of mice tongue exposed to plasma jet was approximately 0.75 cm^2^ (0.5x1.5 cm). Therefore, the applied energy per unit area (discharge mean power times treatment time divided by the tongue area) was calculated as 240 W.s/cm^2^.

To ensure that heating during the plasma treatment was not an issue, temperature on a simulated target (water drop with volume of 0.5 mL and initial temperature of 23°C) was monitored. The sample temperature raised to 37.0 ± 1.5°C for 5 min plasma application using continuous sinewave voltage signal (32.0 GHz and 13.0 kV). Therefore, in this case, sample temperature could reach potentially dangerous limit especially when considering small targets and long treatment time. On the other hand, for amplitude modulated voltage signal with duty cycle of 22% the sample temperature did not exceed 26.0 ± 1.5°C for 5 min of plasma treatment. Therefore, using AM-CAAPJ, there is a substantial reduction of thermal load to the target, which is very important, especially for *in-vivo* applications.

### Effect of He-AM-CAPPJ on *C*. *albicans* biofilms *in vitro*

**Fig 6:**
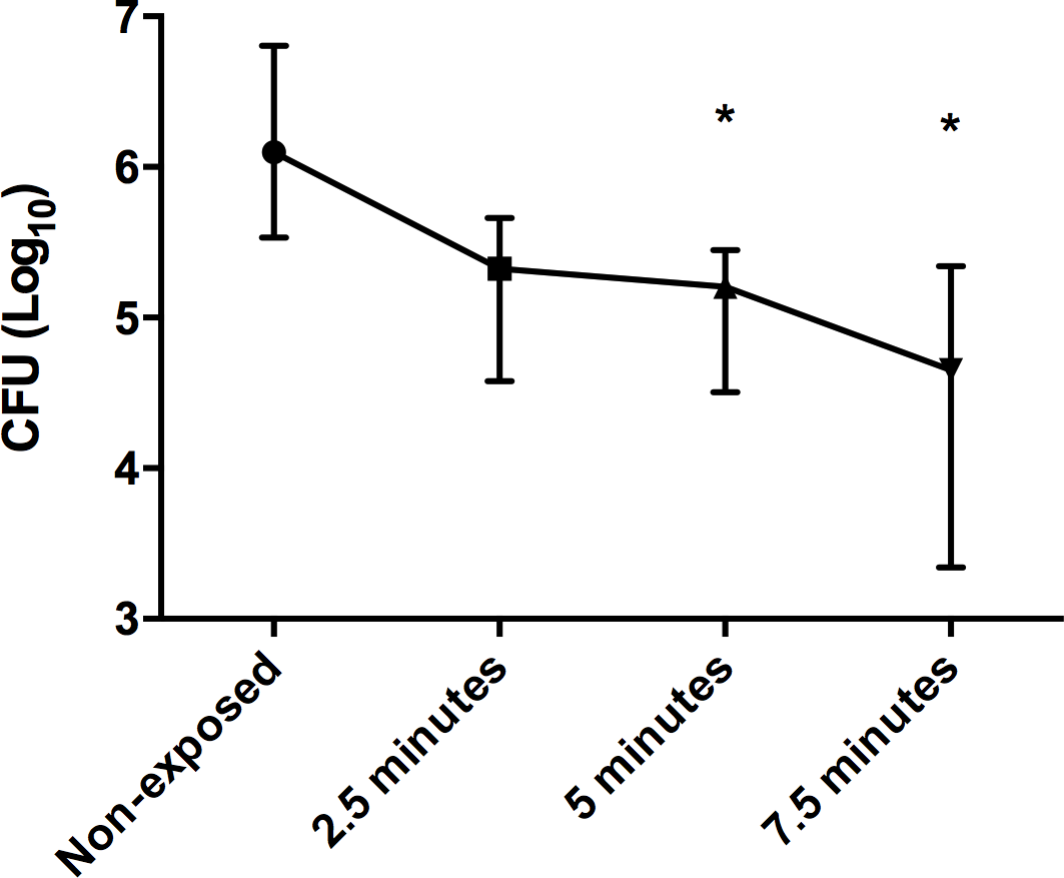
Number of viable cells in *C*. *albicans* ATCC 18804 biofilms (median and range) exposed or not to AM-CAPPJ (n = 9). * p<0.05.

An exposure of 2.5 min to AM-CAPPJ was not able to reduce the number of viable cells in the biofilms (Fig 6). From 5 minutes of exposure, counts of viable cells were significantly lower in the plasma-exposed group compared to the non-exposed group (Kruskal-Wallis/ Dunn’s multiple comparisons test; p = 0.0033). The antibiofilm effects after 5 and 7.5 minutes of exposure were similar (p > 0.05).

### Cytotoxicity of He-AM-CAPPJ *in vitro*

The Vero cell viability immediately after 3 min of exposure to AM-CAPPJ was similar to the control group (100%). The follow up of the cells 24 and 48 hours after exposure suggested the absence of late effects (92.58 ± 6.56% and 98.13 ± 9.25%, respectively).

**Fig 7:**
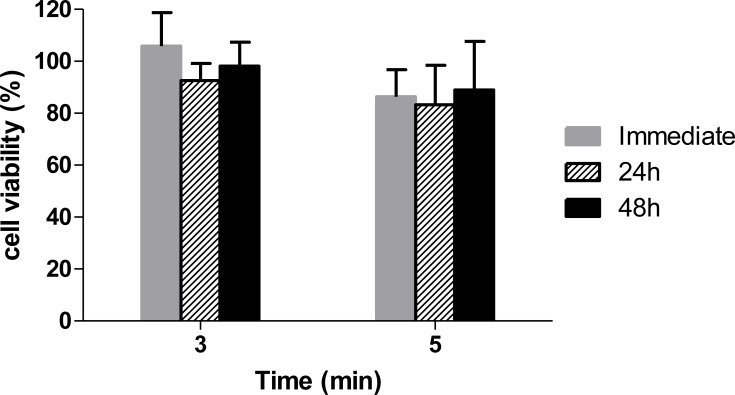
Vero cells viability (mean percentage and standard deviation) after exposure to AM-CAPPJ. Cell viability was measured immediately, 24 and 48 hours after the exposure to plasma jet (n = 6).

After 5 minutes of exposure, high cell viability was still maintained (86.33 ± 10.45%). Even after 5 minutes of exposure, no late effects were observed after 24 and 48 hours (83.24±15.23% and 88.96±18.65%, respectively). Considering that the cell viability was above 70% for all the tested conditions, the results point out to a low cytotoxicity of the plasma jet, both after 3 and 5 min of exposure, as can be observed on Fig 7.

### Treatment of experimentally induced oral candidiasis with AM-CAPPJ

*In vitro* assays showed that the AM-CAPPJ could reduce the viability of *C*. *albicans* in biofilms after 5 minutes of exposure with low cytotoxic effects. Considering these results, 5 minutes of exposure were adopted for the *in vivo* assay.

**Fig 8:**
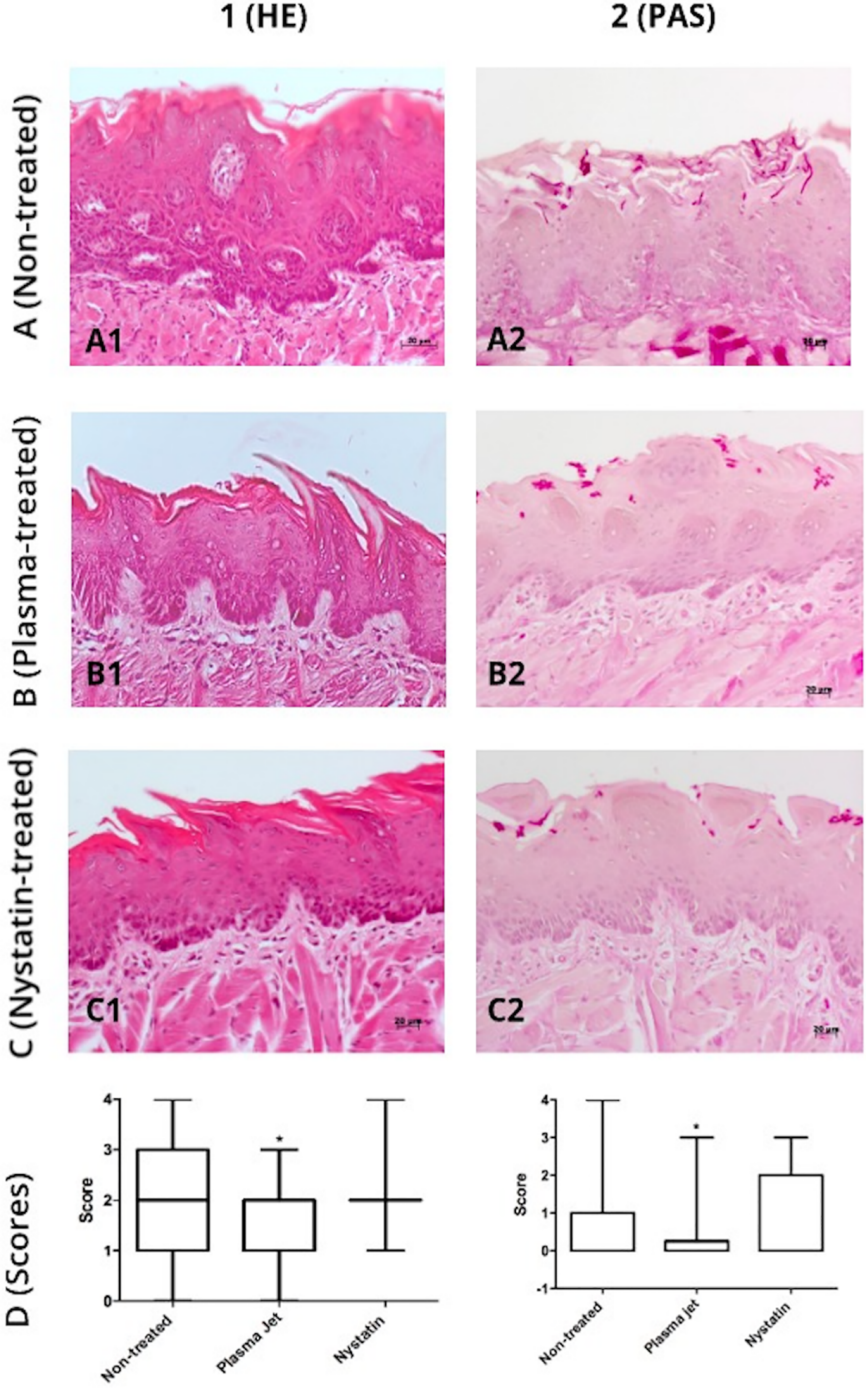
Microscopic images of dorsal surface of *C*. *albicans*-infected tongues 24 hours after AM-CAPPJ exposure. (1) HE-stained; (2) PAS-stained; (A) candidiasis induced and non-treated group; (B) plasma jet-treated; (C) nystatin-treated; (D) Scores (median values and ranges) attributed to the inflammatory alterations (column 1) and tissue invasion (column 2) 24 hours after exposure to AM-CAPPJ.

Descriptive analyses of HE stained slides showed that the non-treated animals exhibited an irregular pattern of the covering epithelium, ranging from hyperplasic to hyperkeratinized. In some cases, the tongue epithelium lost the papillary aspect. A variety of inflammatory alterations were evidenced along the tongue length: hydropic degeneration, spongiosis, basal layer disorganization, exocytosis, and intraepithelial abscess. Underneath the epithelium, an intense and mixed inflammatory cell infiltrate could be seen among the connective tissue fibers, and it became polymorphonuclear predominantly near the muscular layer of the tongue. This infiltrate was more evident at the anterior third of the tongue, where there were large and congested vessels (Fig 8A1). Oral candidiasis was confirmed by the analyses of PAS stained slides (Fig 8A2). It could be observed that candidiasis induced more extended and frequent epithelial alterations in the non-treated group comparing to the other groups.

Tongue fragments of the plasma-treated group (24 hours) presented inflammatory characteristics compatible to oral candidiasis, confirmed by PAS staining (Fig 8B1 and 8B2). These inflammatory alterations associated with the cell infiltrate suggest a light epithelial response to *C*. *albicans*. Hyperplasia, hydropic degeneration, hyperkeratosis and exocytosis were observed. Differently from the control group, the moderate cell inflammatory infiltrate was mononuclear and macrophage-rich with scarce polymorphonuclear cells.

For the nystatin 24-hour group, mice exhibited less inflammatory alterations and hyphae invasion than control group (Fig 8C1 and 8C2). Epithelial hyperplasia and hyperkeratosis, papillary aspect loss, and exocytosis were observed. A moderate mixed inflammatory cell infiltrate was found underneath the epithelium.

The statistical analyses of the scores for inflammatory alterations (Fig 8D1) showed that 24 h after the treatment, the plasma-jet-treated group had significantly lower occurrence of inflammatory alterations (median = 1) when compared to non-treated (median = 2) and nystatin-treated (median = 2) groups (p < 0.0001; Kruskal-Wallis/Dunn).

PAS analyses after 24 h of the last treatment evidenced tissue invasion by *C*. *albicans* hyphae (Fig 8). The plasma jet could reduce *C*. *albicans* tissue invasion, since there was a significant difference in the number of invading hyphae in plasma-treated and non-treated or nystatin-treated groups (p < 0.0001; Kruskal-Wallis/Dunn).

**Fig 9:**
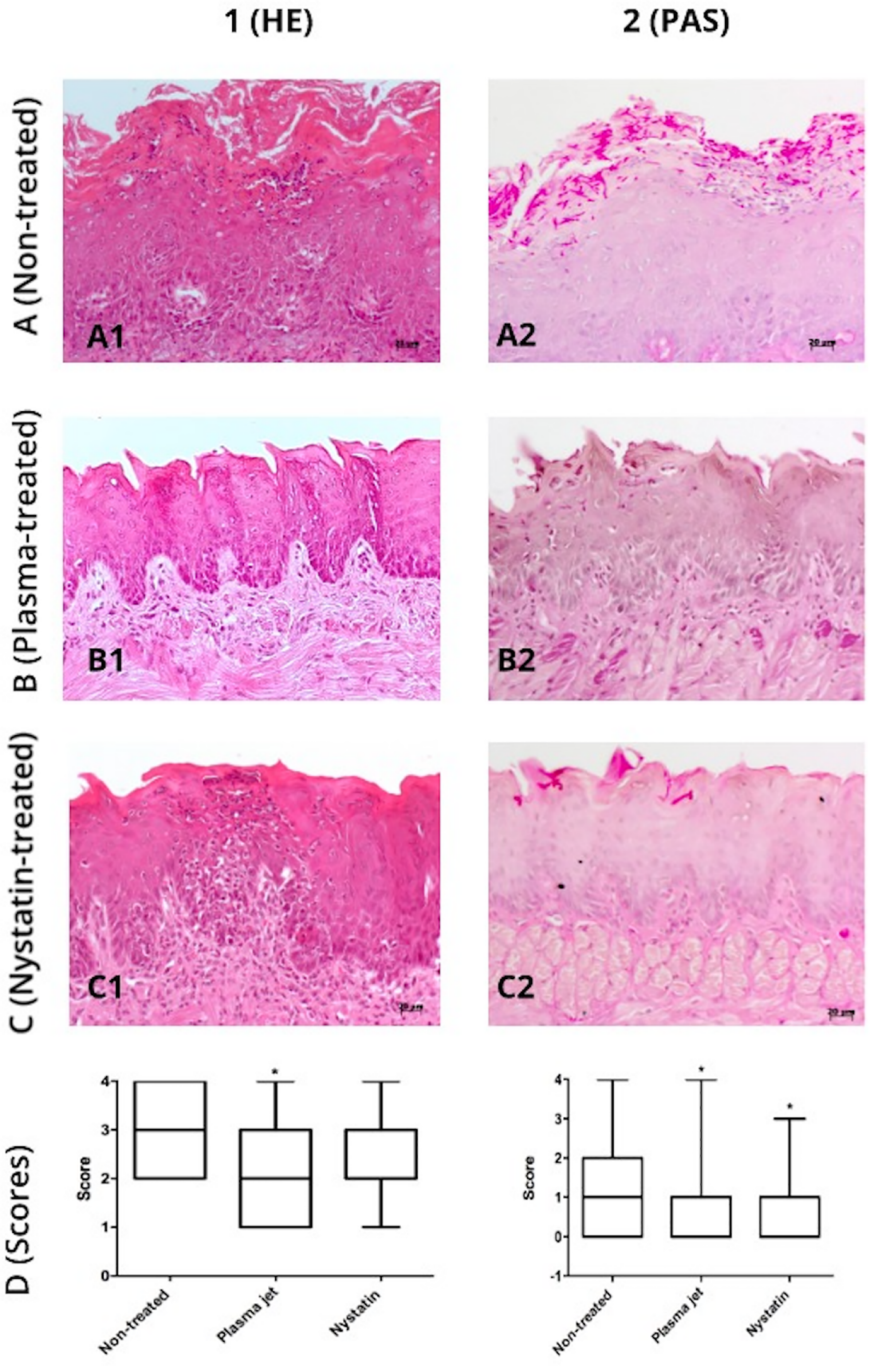
Microscopic images of dorsal surface of *C*. *albicans*-infected tongues 48 hours after AM-CAPPJ exposure. (1) HE-stained; (2) PAS-stained; (A) non-treated group; (B) plasma-treated; (C) Nystatin-treated; (D) Scores (median values and ranges) attributed to the inflammatory alterations and tissue invasion 48 hours after exposure to AM-CAPPJ.

Descriptive analysis of histological sections obtained 48 hours after the treatment showed that the non-treated group showed more intense inflammatory response than the 24-hour group (Fig 9A1 and 9A2). Tongue papillae loss and intraepithelial abscess were common. An intense and mixed inflammatory cellular infiltrate was observed from underneath the histologic candidiasis areas to the deeper connective tissue areas.

On the contrary, when analyzing the 48-hour group treated with AM-CAPPJ, the epithelial inflammatory alterations were slightly more evident than in the 24-hour group. Compared with the other groups, otherwise, they had less inflammatory alterations and inflammatory cell infiltration. Infiltrate was predominantly polymorphonuclear contrasting with the mixed cell infiltrate present in the other groups (Fig 9).

Major epithelial inflammatory alterations were found in histologic candidiasis areas of the nystatin-treated group (48 hours), with remarkable epithelial hyperplasia, loss of tongue papillae and microabscess. The extension and intensity of the epithelial alterations in the nystatin group were smaller than in the control group, as well as the intensity of the inflammatory cell infiltrate (Fig 9).

Forty-eight hours after treatment, the group exposed to the plasma jet showed significant reduced inflammatory alterations when compared to non-treated and Nystatin-treated groups (p < 0.0006; Kruskal-Wallis/Dunn). However, all groups had animals with intraepithelial microabscess associated with other inflammatory alterations (Fig 9).

It was possible to observe 48h after treatment that both treated groups (plasma jet and Nystatin) showed significantly lower number of hyphae invading the tongue epithelium compared to the non-treated group (p < 0.0001; Kruskal-Wallis/Dunn). No difference was observed between treated groups (p > 0.05, Kruskal-Wallis/Dunn), as shown in Fig 9.

Regarding the plasma exposure on non-infected tongues, the histologic sections showed that there was no tissue alteration due to the plasma exposure. The tongue epithelium was intact, with well-defined layers, and papillar aspect preserved. The connective tissue had its integrity preserved as well, with regular fibroblasts, collagen fibers, muscle bindles, and salivary glands. Sparse inflammatory cells were observed.

**Fig 10:**
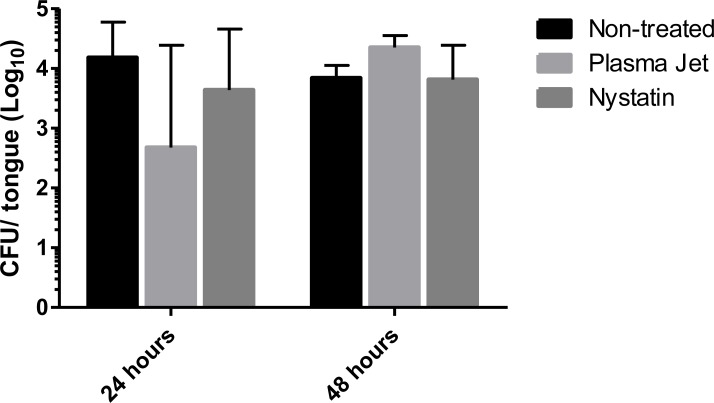
Counts of colony forming units in mice’ tongues 24 hours after the treatment with nystatin or AM-CAPPJ.

No significant differences among the values of CFU/tongue were observed among the control group and the groups treated with plasma jet or nystatin (p = 0.3201; Kruskal-Wallis/Dunn multiple comparisons test), when the animals were sacrificed 24 hours after the last treatment. The same result was observed when the animals were sacrificed 48 hours after the last treatment (p = 0.0616; Kruskal-Wallis/Dunn multiple comparisons test) (Fig 10).

## Discussion

Some studies showed that cold atmospheric plasma jet have anti-*Candida* activity [20, 22, 24]. Recently, Borges et al. [26] showed that cold atmospheric plasma jets operating with Helium and powered with continuous sinewave voltage could disrupt *C*. *albicans* biofilm starting from 300 s exposition period. However, from 150 s of exposure, the plasma jet was also cytotoxic to Vero cells. Thus, the need of a new protocol, simultaneously effective and safe, with real potential clinical application motivated this study. To our knowledge, this is the first study to evaluate the effects of plasma jets for the treatment of experimentally induced oral candidiasis *in vivo*.

A modification of the cold atmospheric pressure plasma jet (CAPPJ) was introduced by using an amplitude modulated voltage signal (AM-CAPPJ) instead of a continuous voltage wave (C-CAPPJ). The working gas was Helium in both protocols. The main reason behind this modification was the reduction of treatment toxicity to mammalian cells by adjusting the discharge power. In the mode of single sinewave voltage waveform, the corresponding discharge power is approximately 2.0 W. When amplitude modulated voltage signal with duty cycle of around 20% is used, the power delivered to the target can be reduced to around 0.5–0.6 W. In this new condition, the target heating is significantly reduced (from 37.0 ± 1.5°C for continuous voltage waveform to 26.0 ± 1.7°C for AM-CAPPJ). As shown by Lu et al. [33] the reactive species are produced not only in the vicinity of HV electrode but also in the region of plasma effluent.

Indeed, the cytotoxicity test by MTT assay showed low cell toxicity after both 3 and 5 minutes’ exposure to AM-CAPPJ, with cell viability higher than 80%. According to the ISO 10993 [29] protocol, medical devices showing cell viability greater than 70% do not present cytotoxic potential.

Toxic effects usually start to appear after longer times of exposure [34–36]. A previous work showed cell viability lower than 45% after 5 minutes of surface barrier discharges with air plasma exposure [36]. Moreover, a study performed by Borges et al. [26] showed cell viability lower than 5% after a 5-minute exposure to Helium plasma jet operating with sinewave voltage signal.

Interestingly, despite of the lower cytotoxicity to Vero cells, the new protocol using AM-CAPPJ showed similar effectiveness against *C*. *albicans* biofilms when compared to the C-CAPPJ [26]. AM-CAPPJ could reduce significantly the number of viable cells in biofilms after 300 s of exposure. Similarly, an exposure of 300 s to O_2_/Ar plasma treatment could disrupt *C*. *albicans* biofilms [21].

The differences in the effect of AM-CAPPJ on mammalian and fungal cells are intriguing, as we expected that the considerable reduction in cytotoxicity to mammalian cells could also result in a proportional reduction in antifungal activity. We hypothesize that the modification in the protocol might cause differences in the concentration of produced reactive species produced that can have different effects on mammalian and fungal cells. Although further investigation can prove this hypothesis, two physical modifications could explain this lower cytotoxicity: the reduction of the power and the amplitude modulation of the plasma device. Besides the reduction of the power in the AM-CAPPJ, it should be noticed that the plasma occurs intermittently, with periods of action interspersed with brief intervals of inactivity, which may alter the kinetics of some reactive species. In turn, this can reduce the action of plasma on mammalian cells while maintaining the activity on fungal cells. Further studies should be performed reducing the power of C-CAPPJ to investigate whether the reduction of the power is the sole responsible for this lower cytotoxicity or the modulation in the amplitude also plays a role.

The results of low *in vitro* cytotoxicity associated to antifungal effectiveness encouraged and supported the *in vivo* experiments.

The application of the plasma jet to the tongue epithelium in non-infected mice reinforced the results of safety suggested by the low cytotoxicity. No tissue alterations were observed after AM-CAPPJ exposure. The structure of epithelial and connective tissues, as well as salivary glands was maintained, and no inflammatory process was observed. Absence of mucosal irritation in rabbits was previously reported by Liu et al. [37] after 10 minutes of exposure to a plasma jet.

The adopted experimental protocol was adequate to induce oral candidiasis. The evolution of the infection was in accordance to the described by previous studies [30, 38] with local acute inflammation and presence of *C*. *albicans* mycelium in superficial tissues reaching the peak 48 hours after the inoculation.

In the current study, we found that plasma was more effective than nystatin after 24 hours in reducing tissue invasion. The effect of plasma and nystatin reaches similar levels after 48 hours from exposure. Additionally, the plasma-jet-treated group showed less inflammatory alterations 24 and 48 hours after the treatment when compared to non-treated and nystatin-treated groups. These findings reinforce the evidences of anti-inflammatory effect of the cold plasma jet [39]. Besides the antimicrobial and anti-inflammatory effects, the plasma jets have antipruritic effect, promote tissue and microcirculation stimulation due to the synergic effect of ultraviolet radiation, electric fields and reactive oxygen species [40].

However, animals with intraepithelial microabscess could be observed in all groups after 48 hours. These results suggest that a multiple-exposure treatment, with exposures to AM-CAPPJ in sequential days, could be more effective to better explore the anti-inflammatory and antifungal activity. This study aimed to evaluate the early stage *C*. *albicans* infection; future studies with longer period of evaluation, multiple-exposure treatment and association between nystatin and AM-CAPPJ should be done to improve the clinical outcomes.

We also noted that there was no significant reduction in *C*. *albicans* CFU counts in the tongue of animals treated with plasma jet or nystatin in comparison to control. However, the histological analyses revealed remarkable reduction in tissue invasion. Interestingly, these findings corroborate the previously reported effect of plasma on candidal morphogenesis [26]. It is important to highlight that the occurrence of disease is result of a complex interplay between the expression of candidal virulence factors, interactions with bacterial microbiome and host’s immune system [3]. The results presented herein, using an experimental model of oral candidosis in immunosuppressed animal, suggest that AM-CAPPJ affects candidal virulence factors, contributing to modulate fungal pathogenicity.

The microenvironment of the mice tongue lesions (infection/inflammatory microenvironment) is complex. On one hand, the immune defense cells also generate ROS [41] in an effort to control the infection. On the other hand, the presence of ROS leads to the production of endogenous antioxidants in order to prevent and repair tissue damages [42, 43]. Also, in vitro studies showed that plasma affects keratinocyte transcriptome and proteome via antioxidant pathways [41]. Endogenous antioxidants may act on the reactive species and a possible attenuation of the plasma activity may occur. In fact, previous studies reported that antioxidants present in the sample can affect plasma effectiveness [32, 44]. The extent of this attenuation and overall influence on plasma activity with our device has yet to be determined in future investigations.

In conclusion, these results are promising and point out to a clinical applicability of this protocol, due to the simultaneous anti-inflammatory and inhibitory effects on candida morphogenesis of AM-CAPPJ with low cytotoxicity.
